# The association between pre-pregnancy and first-trimester hair cortisol and preterm birth: a causal inference model

**DOI:** 10.1007/s10654-024-01174-w

**Published:** 2024-12-11

**Authors:** Yinxian Chen, Richard G. Künzel, Sixto E. Sanchez, Marta B. Rondon, Nelida I. Pinto, Elena Sanchez, Clemens Kirschbaum, Linda Valeri, Karestan C. Koenen, Bizu Gelaye

**Affiliations:** 1 Department of Epidemiology, Harvard T.H. Chan School of Public Health, Boston, MA 02115 USA; 2 Department of Epidemiology, Rollins School of Public Health, Emory University, GA 30322 Atlanta, USA; 3 Katholische Universität Eichstätt-Ingolstadt, 85072 Eichstätt, Germany; 4 Facultad de Medicina Humana, Universidad de San Martin de Porres, Instituto de Investigación, Lima, 15024 Perú; 5 Asociación Civil Proyectos en Salud, Lima, 15024 Perú; 6 Universidad Peruana Cayetano Heredia, Lima, 15024 Perú; 7 Technische Universität Dresden, 01069 Dresden, Germany; 8 Department of Biostatistics, Columbia University Mailman School of Public Health, New York, NY 10032 USA; 9 Department of Psychiatry, The Chester M. Pierce M.D. Division of Global Psychiatry, Harvard Medical School, Massachusetts General Hospital, Boston, MA USA

**Keywords:** Hair, Cortisol, Cortisone, Corticosteroid, Preterm birth, Causal inference

## Abstract

Adverse life events and chronic psychological distress before and during pregnancy have frequently been associated with preterm birth but the biological underpinnings remain unclear. We investigated the association between corticosteroid levels in pre-pregnancy and first-trimester hair and the risk of preterm birth. We followed *N* = 1,807 pregnant women from a prospective pre-birth cohort study in Lima, Perú. Hair samples were taken at the end of the first pregnancy trimester. The two most proximal 3 cm segments to the scalp (representing pre-pregnancy and first-trimester) were analyzed to obtain hair cortisol and cortisone concentrations (HCC and HCNC). Preterm birth was defined as birth < 37 completed gestational weeks. We constructed four generalized propensity scores for pre-pregnancy and first-trimester HCC and HCNC to create corresponding inverse probability weights before fitting marginal structural models for estimating the effect of HCC and HCNC on preterm birth risk. Pre-pregnancy Log HCC was not independently associated with preterm birth risk (RR = 0.97; 95%CI: 0.79, 1.19), nor was pre-pregnancy Log HCNC (RR = 0.84; 95%CI: 0.58, 1.20). In the first trimester, a one SD increase in Log HCC was associated with a 37% increased risk of preterm birth (95%CI: 1.11, 1.69), whereas Log HCNC was not significantly associated with preterm birth risk (RR = 1.20; 95%CI: 0.87, 1.65). Our findings show that chronic corticosteroid levels in early pregnancy are causally linked to preterm birth risk in pregnant Peruvian women. This finding contributes to understanding the biological underpinnings of preterm birth better to enhance its prevention.

**Supplementary Information:**

The online version contains supplementary material available at 10.1007/s10654-024-01174-w.

## Introduction

Preterm birth remains one of the most pressing and unsolved issues in public health [1], accounting for 18–28% of childhood mortality until the age of 5 years [2, 3]. Despite improvements in preterm birth prevention [4], approximately 13 million children are born prematurely worldwide every year [1] with disproportionally higher rates in low and middle-income countries compared to high-income countries [1]. Preterm birth can have severe life-course consequences for both mother and child. For example, children born preterm face an increased risk for cardiovascular and renal disease [5], diabetes [6], asthma [7] and neurodevelopmental impairment [8], among other conditions [9]. Accordingly, women who deliver preterm have a higher risk for cardiovascular disease than those who deliver at term [10]. Likewise, preterm birth has been associated with an elevated risk for maternal psychopathology including postpartum depression [11, 12]. Although the specific etiology of preterm birth remains unknown, an increased risk for preterm birth has been associated with family and obstetric history of preterm birth [13, 14], maternal pre-pregnancy health status [15, 16], but also social and structural disparities, such as racism and historical redlining [17, 18], among others [19]. Furthermore, the effect of psychological distress on preterm birth is a rapidly evolving area of investigation as a number of studies, including our own, have found associations between symptoms of mood [20–25] and anxiety disorders [23, 24, 26–29] and elevated risk for preterm birth, although not consistently [26, 30, 31]. Additionally, previous research has identified psychologically distressing events, such as early life adversity or intimate partner violence, as risk factors for preterm birth [32–34], which are of a high prevalence in many low- and middle-income countries (LMICs) and often precede symptoms of psychopathology [35, 36].

The specific biological underpinnings between symptoms of psychological distress and preterm birth risk also remain unknown. Nevertheless, the hypothalamic-pituitary-adrenal (HPA) axis has been suggested as a possible biological pathway, as it is the body’s primary stress-response system [37]. In response to stressful experiences, the hypothalamus gets stimulated, secreting the corticotropin-releasing hormone (CRH) [38, 39]. CRH acts stimulating on the pituitary, that secretes the adrenocorticotropic hormone (ACTH) into the blood stream, which targets the adrenal cortex [40]. The adrenal cortex then secrets glucocorticoids and catecholamines [40], of which cortisol is the most important for HPA axis functioning [41]. Through the enzyme 11β-HSD-2, the biological active cortisol gets metabolized into biologically inactive cortisone [42]. Various studies have shown that cortisone and cortisol are highly correlated in saliva and hair specimens [43–45]. Given that the concentration of cortisone is higher in saliva and hair compared to cortisol, and it is less influenced by environmental factors, cortisone has been suggested to be a potentially more robust biomarker of systemic HPA axis regulation than cortisol [43]. Interestingly, cortisol is also involved in pregnancy regulation, as findings of increasing average cortisol concentrations across pregnancy, measured with varying biospecimen, suggest [46, 47]. Human and animal studies indicate that cortisol and its related hormones are involved in labor onset [48], as well as fetal brain and lung development [49, 50]. Cortisol and cortisone can be measured in urine, blood and saliva, although situational and diurnal variations limit their use for long-term evaluation of HPA axis activity [51]. Therefore, cortisol and cortisone assessments in human scalp hair have emerged in recent years as novel tools to assess long-term hormone secretion [52]. Because hair growth is relatively stable over time [53, 54], hair cortisol concentration (HCC) and hair cortisone concentration (HCNC) indicate the respective hormone secretion over a specific timespan [55], such as pregnancy. Hence, HCC and HCNC have become promising biomarkers of psychological distress in both, pregnant and non-pregnant individuals [56, 57] and respective correlations with preterm birth have been shown in previous studies [28, 58–64].

However, previous studies have solely conducted correlational analyses predominantly with samples from high income countries, that yielded inconclusive results regarding the predictive value of HCC and HCNC for preterm birth [65], albeit the urgent need to identify the potential causal role chronic cortisol and cortisone play in preterm birth etiology. Furthermore, previous research noted that baseline cortisol levels can be chronically altered depending on a history of stressful and traumatic experiences, raising the question of whether such pre- and early pregnancy HCC alterations could impact the pregnancy course and outcomes [66]. Given that both the lifetime stress burden among young women [67, 68] and preterm birth prevalence [1] are often elevated in LMIC in the global south, examining this relationship in these understudied regions can enhance our understanding of its cultural and regional dimensions.

Therefore, in the present study we aimed to i) identify the independent causal relationship between HCC, HCNC and preterm birth risk and ii), investigate the cumulative effect of HCC and HCNC at different pregnancy time points on preterm birth risk in a large sample of an understudied population.

## Methods

### Study population

The sample for this study was drawn from participants of the Pregnancy Outcomes, Maternal, and Infant Study (PrOMIS), a prospective cohort study with the objective to identify maternal, social and behavioral risk factors for adverse pregnancy outcomes in Lima, Perú (*N* = 5440). Participants were women attending prenatal care clinics of the Instituto Nacional Materno Perinatal (INMP), which serves as the principal establishment for maternal and prenatal care in Perú. Study methodology has been reported previously [69]. Participants were recruited between February 2012 and November 2015 and followed up until delivery. Participants were eligible if they initiated prenatal care prior to the 16th gestational week [69], were ≥ 18 years old, were able to speak and read Spanish, had a singleton pregnancy, and intended to deliver at the INMP. Written informed consent was obtained from all participants. All study procedures were approved by the institutional review boards of the INMP, Lima, Perú and the Harvard T.H. Chan School of Public Health, Boston, MA, USA. In this study, participants with two measures in at least one type of hair corticosteroid concentration and a record of gestational age at birth were included in the study population.

### Data collection

#### Sociodemographic, reproductive and covariate data

Sociodemographic, medical and reproductive history and hair-related data were collected through structured interviews conducted by trained research personnel at the time of recruitment. The sociodemographic data included maternal age (years), marital status (married or live with partner/ other), ethnicity (mestizo/ non-mestizo), employment during pregnancy (yes/ no) and difficulty paying for basic items such as food (yes/ no). Pre-pregnancy and first-trimester weight (kg) and height (m) were collected to the nearest 0.1 kg and 0.1 cm to calculate the respective body mass index (BMI; kg/m^2^). Serum C-reactive protein (mg/pg) was measured from women’s non-fasting blood samples at the time of enrollment as an inflammatory marker. Details of the measure of serum C-reactive protein in the PrOMIS study were previously described [70]. Gestational age upon registration was determined by self-report of the last menstrual period and confirmed by ultrasound examination prior to 20 weeks of gestation. Hair-related information at data collection included hair washing frequency (1–2 times/week, 3–5 times/week, 6–7 times/week), hair dye (yes/ no), and hair tint (yes/ no). Medical and reproductive history information included planned pregnancy (yes/ no), parity (nulli-/ multiparous) and infant sex (male/ female). After delivery, medical records of the mother and newborn were abstracted to assess the pregnancy course and outcomes, including preterm birth. We defined preterm birth as delivery prior to completed gestational week 37, according to guidelines by the American College of Obstetricians and Gynecologists (ACOG) [71] and categorized preterm birth cases according to three pathophysiological groups (spontaneous preterm birth, preterm premature rupture of membranes, and medically induced preterm birth). Spontaneous preterm birth cases comprised women with a physician diagnosis of spontaneous labor onset (with intact fetal membranes) and delivery prior to the completion of 37 weeks’ gestation, as indicated by medical records. Preterm premature rupture of membranes cases comprised women with a physician diagnosis of rupture of fetal membranes (prior to the onset of labor) and delivery prior to the completion of 37 weeks’ gestation, as recorded in medical records. Women who delivered before completed gestational week 37 due to medical intervention were excluded from this study.

#### Hair collection procedures

Full-length hair samples were collected by trained research personnel from the posterior vertex region of the head at the end of the first pregnancy trimester. Hair was cut as close to the scalp as possible and cut into three 3 cm segments. Thus, each segment reflected approximately 3 months, based on an average growth rate of 1 cm/month [53, 54]. Only the two segments closest to the scalp were analyzed due to possible washout effects, resulting in less reliable measurements of corticosteroids in the most distal hair segment [55, 72]. Hence, the proximal 3 cm segment reflected the first trimester of pregnancy, and the distal segment reflected 0–3 months pre-pregnancy. Due to hair length limitations, *n* = 16 participants (0.9% of the analytic sample) could not provide hair longer than 3 cm. Given the need for complete data on both segments in the statistical analyses, these participants were excluded from the analysis. Their sociodemographic characteristics did not differ from the analytic sample. After collection, hair samples were wrapped in aluminum foil, stored in envelopes away from light, and kept at room temperature using desiccant. Hair samples were sent to the Kirschbaum laboratory at Dresden University of Technology, Germany, for analysis.

#### Laboratory analysis

First, hair samples were washed twice in 2.5 ml isopropanol for three minutes in a 15-ml falcon tube and dried for 12 h. Second, 7.5 mg of whole non-pulverized hair was weighed out per segment and minced into small pieces. This procedure was chosen according to in-house experiment results by the executing lab [73]. Third, at room temperature, hair samples were incubated in 1.8 ml of high-grade methanol for 18 h. Fourth, 1.6 ml of clear supernatant was transferred to a vial. Fifth, the methanol was evaporated off for 30 min at 55 °C using nitrogen and then resuspended in 225 µl distilled water before adding 20 µl internal standard (cortisol-d4). Sixth, HCC and HCNC levels were quantified by liquid chromatography tandem mass spectrometry (LC-MS/MS) assay using 50 µl of the total resuspension. The LC-MS/MS had a lower detection limit of 0.1 pg/mg. *N* = 1 participant had to be excluded from the analysis due to undetectable corticosteroid concentration. The intra- and inter-assay coefficient of variation were 11.9% and 19.4%, respectively, which is within acceptable ranges. Hair segments from the same participant were assayed together in the same batch to minimize batch-to-batch variability. HCC and HCNC units were expressed in picograms per milligram (pg/mg).

### Statistical analysis

Descriptive statistics, where mean (SD) was used for continuous variables and frequency (%) for categorical variables, were displayed for characteristics of the study population. A comparison of characteristics between the study and non-study population from the PrOMIS study was performed. The mean (SD) and median (IQR) were also summarized for HCC and HCNC in pre-pregnancy and first trimester, and the correlations between hair corticosteroid levels in different periods were calculated using Spearman correlation.

Before we performed the causal association assessment with full measures of HCC or HCNC, we trimmed each sub-population at 1 and 99 percentiles of HCC or HCNC to minimize the impact of extreme values on estimating the association. Since the missing rate in each variable was < 5% (Table S1), the primary analysis was focused on samples with complete data on all included covariates (i.e., the complete case analysis). Pre-pregnancy and first-trimester HCC and HCNC were transformed into the natural log scale due to their skew distributions in the population before being standardized to mean = 0 and SD = 1. First, a total of four generalized propensity scores (GPS) were constructed for pre-pregnancy and first-trimester HCC and HCNC using the generalized additive model (GAM). Each model contained penalized regression spline terms for all continuous predictors to account for their potential non-linear association with hair corticosteroid concentration. The covariates for each propensity score model were selected if they were potential confounders based on their hypothetical association with hair corticosteroid concentration and preterm birth (Fig.S1). Second, we utilized stabilized inverse-probability weights (SIPW) calculated by GPS to construct marginal structural models (MSM) using Poisson regression to estimate the independent causal effect of HCC and HCNC on two occasions with risk of preterm birth, respectively (Table S2). We reported relative risks (RRs) for each occasion and 95% confidence intervals (CIs) calculated by robust standard errors. The cumulative effect of HCC and HCNC across time with preterm birth risk could then be calculated by multiplying RRs on two occasions. To examine whether the SIPW created by GAM-estimated GPS was successful in addressing confounding, we compared the unweighted correlation between hair corticosteroid concentration and each potential confounder with the correlation weighted by SIPW. We computed the average absolute correlation with and without SIPW to display the overall performance of achieving covariate balance and balance plots to illustrate the performance for each covariate. The method for calculating the correlation has been previously described [74, 75].

Secondary analyses were focused on: (1) testing whether the cumulative association of HCC and HCNC with preterm birth risk would vary depending on the levels of HCC and HCNC at different times by comparing MSM with and without the product term “Pre-pregnancy*First trimester”, and (2) testing the potential curvilinearity in the association of HCC and HCNC with preterm birth risk by fitting MSM using GAM with penalized spline terms and comparing with the original MSM in primary analysis. All tests were performed by the Likelihood ratio test.

Several sensitivity analyses were performed. First, we addressed the missing values by employing multiple imputation using the *mice* package in R (Appendix S1) and reran the primary analysis. Second, we fit the GPS using multivariate linear regression instead to create SIPW. We also compared the weighted average absolute correlation, as well as balance plots, by different GPS fitting approaches (i.e., GAM vs. linear regression). Third, we additionally included serum C-reactive protein when fitting the GPS for the first-trimester HCC and HCNC to account for potential confounding by inflammation and reran the primary analysis.

## Results

### Descriptive results

This study included *N* = 1,807 pregnant women (33% of the PrOMIS study), with the demographic characteristics and outcome distribution described in Table 1. The mean age of participants was 28.0 (SD = 6.2) years. The majority of participants reported being married or living with a partner (83%), being of mestizo ethnicity (80%) and being pregnant unplanned (59%). Approximately half of the participants reported employment during pregnancy (49%), being nulliparous (47%) and having difficulty paying for basics such as food (45%). BMI did not substantially vary, with the mean BMI shifting from 25.5 (SD = 4.0) kg/m^2^ before pregnancy to 25.7 (SD = 4.1) kg/m^2^ at the recruitment visit during pregnancy. Regarding hair treatment, most of the women (71%) reported a hair wash 3–5 times/ week, and hair tint (43%) was more common than hair dye (15%). The mean gestational age during the hair samples collection was 14.8 (SD = 7.5) weeks. The cumulative incidence of spontaneous preterm birth in this population was 7% during the follow-up, and male infants accounted for 51% of the total births. There was no substantial difference in characteristics between the study and non-study population (Table S3).

**Table 1:** Characteristics of pregnant women sample. Lima, Perú. *N* = 1,807

Characteristics	*n* (%) ^a^
Maternal age (year), mean (SD)	28.0 (6.2)
Maternal age (year) category	
18–19	101 (5.6)
20–29	1,008 (55.8)
30–34	393 (21.8)
≥35	304 (16.8)
Difficulty in paying for basics	
No	987 (54.9)
Yes	811 (45.1)
Employment during pregnancy	
Unemployed	913 (50.6)
Employed	892 (49.4)
Marital status	
Others	311 (17.3)
Married or live with a partner	1,491 (82.7)
Nulliparity	
No	959 (53.2)
Yes	842 (46.8)
Ethnicity	
Not Mestizo	354 (19.6)
Mestizo	1,450 (80.4)
Planned pregnancy	
Unplanned	1,065 (59.3)
Planned	731 (40.7)
Gestational age at hair collection, mean (SD)	14.8 (7.5)
Infant sex	
Male	908 (51.2)
Female	867 (48.8)
Pre-pregnant BMI (kg/m^2^), mean (SD)	25.5 (4.0)
Pre-pregnant BMI category	
<18.5	26 (1.4)
18.5–24.9	885 (49.2)
25.0-29.9	657 (36.6)
≥30	229 (12.7)
BMI at pregnancy (kg/m^2^), mean (SD)	25.7 (4.1)
BMI category at pregnancy	
<18.5	32 (1.8)
18.5–24.9	820 (45.7)
25.0-29.9	695 (38.8)
≥30	246 (13.7)
Preterm birth (gestational age < 37 weeks)	
No	1,676 (92.8)
Yes	131 (7.2)
Hair dye	
Yes	261 (14.7)
No	1,520 (85.3)
Hair tint	
Yes	761 (42.7)
No	1,021 (57.3)
Hair wash	
1–2 times/week	68 (3.8)
3–5 times/week	1,270 (71.2)
6–7 times/week	445 (25.0)

Abbreviations: BMI, body mass index; SD, standard deviation

^a^The number of participants in each variable may not be identical to the total sample sizes due to missingness

Among the *N* = 1,807 individuals included in the study, *n* = 1,799 had data on HCC, *n* = 1,661 on HCNC. As described in Table 2, the HCC and HCNC increased in the first trimester of pregnancy compared with the pre-pregnancy levels. However, the increase was more pronounced in HCNC compared with HCC. HCC and HCNC during pre-pregnancy and the first trimester were moderately to strongly correlated with each other (Fig. S2).

**Table 2:** Characteristics of hair corticosteroid levels of pregnant women during pre -pregnancy and first trimester of pregnancy. Lima, Perú

	Hair segment	
	Pre-pregnancy	First trimester
**HCC (pg/mg)**	*N* = 1,799	
Mean (SD)	4.12 (5.10)	5.10 (6.54)
Median (IQR)	2.83 (2.67)	3.57 (3.26)
**HCNC (pg/mg)**	*N* = 1,661	
Mean (SD)	5.33 (5.85)	9.95 (9.50)
Median (IQR)	3.74 (3.69)	7.38 (6.41)

Abbreviations: HCC, hair cortisol concentration; HCNC, hair cortisone concentration; IQR, interquartile range; SD, standard deviation

### Primary analysis

As shown in Table 3 there was no evidence of association between mean pre-pregnancy Log HCC and the risk of preterm birth (RR = 0.97, 95% CI: 0.79, 1.19). Correspondingly, pre-pregnancy Log HCNC was not significantly associated with the risk of preterm birth (RR = 0.84, 95%CI: 0.58, 1.20).

Regarding the first trimester, a one SD increase from the mean first-trimester Log HCC was independently associated with a 37% increased risk of preterm birth (95%CI: 1.11, 1.69). In contrast, a one SD increase in the first-trimester Log HCNC was not associated with the risk of preterm birth (RR = 1.20, 95%CI: 0.87, 1.65).

**Table 3:** Marginal association between hair corticosteroid levels and risk of preterm birth

	Pre-pregnancy	First trimester
	RR (95%CI)	RR (95%CI)
Log HCC^a^ (*N* = 1647)	0.97 (0.79,1.19)	1.37 (1.11,1.69)
Log HCNC^a^ (*N* = 1520)	0.84 (0.58,1.20)	1.20 (0.87,1.65)

Abbreviations: HCC, hair cortisol concentration; HCNC, hair cortisone concentration

^a^ Log HCC and Log HCNC have been standardized (mean = 0 and SD = 1)

### Secondary analysis

Table 4 shows that the cumulative association of Log HCC with preterm birth risk did not vary by the level of pre-pregnancy and first-trimester Log HCC (*b* = -0.01, *P* = 0.89). There was no evidence that the cumulative association between Log HCNC and preterm birth risk varied by the level of pre-pregnancy and first-trimester Log HCNC (*b* = -0.12, *P* for interaction = 0.20). Furthermore, little evidence shows that Log HCC and Log HCNC were associated with preterm birth risk in a non-linear pattern (Fig. S3, Fig. S4).

**Table 4:** Test for interaction between hair corticosteroid levels on different occasions in relation to risk of preterm birth

	Pre-pregnancy	First trimester	Pre-pregnancy*First trimester	
	RR (95%CI)	RR (95%CI)	*b*	*P* ^b^
Log HCC (*N* = 1647)	0.97 (0.77,1.23)	1.37 (1.12,1.68)	-0.01	0.89
Log HCNC (*N* = 1520)	0.83 (0.58,1.19)	1.19 (0.88,1.63)	-0.12	0.20

Abbreviation: HCC, hair cortisol concentration; HCNC, hair cortisone concentration

^a^ Log HCC and Log HCNC have been standardized (mean = 0 and SD = 1)

^b^*P* was derived from the Likelihood-ratio test comparing the model with to without the interaction term (Pre

pregnancy*First trimester)

### Covariate balance

Table 5 shows that SIPW created by GAM-estimated GPS successfully made Log HCC and Log HCNC independent of potential confounders. Weighted average absolute correlation was substantially dropped compared with unweighted average absolute correlation in both Log HCC and Log HCNC across occasions. Fig. S5 and Fig. S6 show that SIPW shrank each absolute correlation between Log HCC, as well as between Log HCNC, and potential confounder to lower than 0.1, indicating that it had a satisfactory performance in achieving covariate balance.

### Sensitivity analysis

Table S4 shows that estimates from the imputed analysis were highly consistent with the complete case analysis. By using linear regression to fit GPS and create SIPW, the association estimates were concordant with the estimates from using GAM (Table S5). The weighted average absolute correlation by linear regression and GAM were almost identical (Table S6), and the performance of shrinking each absolute correlation was similar (Fig. S7 and S8). Including serum C-reactive protein when fitting the GPS for the first-trimester Log HCC and HCNC yielded consistent results with the primary analysis (Table S7).

**Table 5:** Average absolute correlation between hair corticosteroid levels and each covariate without and with IPW

	Pre-pregnancy		First trimester	
	Unweighted AAC	Weighted AAC	Unweighted AAC	Weighted AAC
Log HCC (*N* = 1647)	0.042	0.008	0.061	0.026
Log HCNC (*N* = 1520)	0.067	0.013	0.073	0.048

Abbreviation: AAC, average absolute correlation; HCC, hair cortisol concentration; HCNC, hair cortisone concentration

## Discussion

To shed further light on the causal role of chronic corticosteroids in preterm birth etiology, our study examined the effect of pre- and early pregnancy corticosteroid levels on preterm birth risk in a large sample of Peruvian women. We found that an independent increase of HCC in the first trimester was associated with an increased preterm birth risk, whereas pre-pregnancy HCC and HCNC at any timepoint was not associated with preterm birth risk.

Whether the mental and physiological state of stress regulation before pregnancy affects the pregnancy course and its outcomes is an ongoing debate [76]. In our study, we found no evidence of an independent association between pre-pregnancy HCC and HCNC and the risk of preterm birth. This aligns with our earlier findings form a study of *N* = 137 pregnant women from Perú, in which we also found no statistically significant association between pre-pregnancy HCC and the risk of preterm birth [59]. However, the direction of the non-significant estimates in both studies was concordant, namely negative. Despite this concordance, the null association between pre-pregnancy corticosteroids and preterm birth found in this study reflects the current state of the literature well. Although there is overall agreement that corticosteroid levels are reliable indicators of “general stress-related processes” [77], ambiguity remains regarding the specific types and the temporality of stress that corticosteroid levels represent [77]. On the one hand, long-term HCC alterations have been linked with psychological distressing experiences. For example, in our own recent study we found that the experience of a traumatic event, such as child abuse, is associated with pre-pregnancy HCC and HCNC [86]. On the other hand, several studies report clearer associations between HCC and recent or ongoing psychological distress, compared to long-term alterations [77]. This duality in the literature may help explain the mixed findings on the associations between self-reported lifetime stress and preterm birth [78–80]. While our findings do not convey evidence of an independent association between stress-related pre-pregnancy corticosteroids and preterm birth risk, an influence of pre-pregnancy corticosteroid levels on the pregnancy course and outcomes should not be completely ruled out. For instance, pre-pregnancy corticosteroid levels may influence pregnancy outcomes by affecting the trajectory of corticosteroid secretion in later pregnancy – a speculation beyond the scope of our study. Further research is needed to explore these potential alternative mechanisms.

Regarding the first trimester, we found an elevated risk of preterm birth for increases in the first-trimester HCC. This finding is partly in agreement with findings from our own recent metal-analysis of *N* = 9 studies, in which we found a higher first-trimester HCC among preterm-delivering women, compared to term-delivering women, although not statistically significant [65]. Inconsistencies with null findings from some included studies are likely due to differences in the study population and low statistical power [65]. In light of our null findings at pre-pregnancy, we speculate that women with excessive HCC elevations in the first trimester may have experienced stress during that period, compared to pre-pregnancy. Similar temporally or condition-specific associations have been observed in related studies, too. For example, traumatized individuals often show a blunted cortisol response to social stress in a laboratory setting compared to non-traumatized individuals [81–83] but display elevated cortisol levels when faced with trauma-related conditions, such as trauma recall or confrontation [84, 85]. Given that almost 60% of our participants reported an unplanned pregnancy, 70% child abuse, and 30% intimate partner violence [86, 87], we suppose that the increased preterm birth risk associated with first-trimester corticosteroid elevations may be driven by an elevated pregnancy-related stress response among these traumatized individuals.

When examining the cumulative effect of chronic corticosteroids on preterm birth risk, we found no effect of cumulative HCC or HCNC on preterm birth risk. This further highlights the temporal specificity of the potential effects of chronic corticosteroid concentration on preterm birth risk. Drawing on the contradictory findings of previous studies [65], finding both, higher [60, 62] and lower corticosteroids [28, 59, 61] in relation to preterm birth risk, we investigated potential non-linear associations between corticosteroid levels and preterm birth risk. Indeed, we found tentative evidence for a non-linear linkage. However, further studies are needed to allow for biological interpretations of this finding. The exclusivity of significant associations to HCC, but not HCNC, is somewhat surprising. Both biomarkers are suggested to reflect stress-related HPA axis regulation, with HCNC having a higher concentration and being less affected by potential synthetic glucocorticoid contaminations [45]. Given that synthetic glucocorticoid use in our sample was unlikely given the assessment at early pregnancy, and sensitivity analyses showed robust results after additional adjustment for an inflammatory marker, we consider our HCC findings as robust. Nevertheless, more research is needed to understand the differential effects of both hormones better.

The results of our study provide new insights into the causal effect of temporal chronic corticosteroid levels on preterm birth risk, implicating that the corticosteroid level an individual has in first trimester may significantly shape the pregnancy course and its outcomes. However, there are some limitations to consider. First, under the current causal inference methodology, potential misspecification of the models used for creating the propensity scores and subsequent IPWs cannot be ruled out. Additionally, the assumption of no unmeasured confounders in this study might be challenged since this is an observational study with residual confounding that cannot be accounted for. Nevertheless, we included as many covariates as possible that might confound the causal effect of corticosteroid levels on preterm birth risk to try to eliminate the influence of residual confounding. Second, although we used the multiple imputations technique to adjust for the underlying bias caused by missingness at random, the bias by potential missingness not at random could not be addressed. However, since the total missingness rate in this study is < 5%, the influence of missingness not at random seems unlikely. Third, our findings may not be generalizable to other populations. Although investigating pregnant women in Perú contributed to mitigating the imbalance between studies in high versus low- and middle-income countries, the particularly high exposure of study participants to life stressors such as poverty, household violence and abuse makes our study results unique. Finally, our study scope was limited to corticosteroid assessments to pre-pregnancy and first trimester as we intended to examine the effect of corticosteroid levels around the time of conception on pregnancy outcomes. Future studies are needed to validate our findings and to expand this scope to the remaining pregnancy course.

## Conclusion

Being born too soon can detrimentally affect health throughout the life course. To mitigate this detrimental effect, an understanding of the causal underpinnings of preterm birth is crucial. In our study of a large sample of pregnant women from Perú, we found that higher chronic corticosteroid levels in the first pregnancy trimester were associated with an increased risk of preterm birth, whereas higher levels during pre-pregnancy seem not to influence preterm birth risk independently. Furthermore, non-linearity of this association and sample-specific effects of corticosteroid levels at different time-points seem possible. This linkage is a promising pathway to better understanding the pathogenesis of preterm birth.

## Electronic Supplementary Material

Below is the link to the electronic supplementary material.

Supplementary Material 1
