# Supplementary material for: The association between pre-pregnancy and first-trimester hair cortisol and preterm birth: a causal inference model
Source: Eur J Epidemiol. 2024 Dec 11;39(12):1391–400. doi: 10.1007/s10654-024-01174-w (PMC11680651; doi:10.1007/s10654-024-01174-w)
Supplement: Supplementary file 1 — Supplementary Material 1 [file 10654_2024_1174_MOESM1_ESM.docx]

1. **Supplementary Material**

**Table S1**. Missingness in selected covariates (*N* = 1,807)

| **Covariate** | **Frequency of missingness (%)** |
| --- | --- |
| Maternal age | 1 (0.1) |
| Difficulty accessing basic foods | 9 (0.5) |
| Employment during pregnancy | 2 (0.1) |
| Marital status | 5 (0.3) |
| Nulliparity | 6 (0.3) |
| Race | 3 (0.2) |
| Planned pregnancy | 11 (0.6) |
| Gestational age at hair collection | 1 (0.1) |
| Infant sex | 32 (1.8) |
| Hair dye | 26 (1.4) |
| Hair tint | 25 (1.4) |
| Hair wash | 24 (1.3) |
| Pre-pregnant BMI | 10 (0.6) |
| BMI at pregnancy | 14 (0.8) |

**Appendix S1.** Description of multiple imputation

The imputations for missing covariates were conducted among participants with complete records of hair corticosteroid concentration and status of preterm birth. Multivariate Imputation by Chained Equation (MICE) was applied separately when assessing different indicators of corticosteroids. All variables in this study, including the outcome (i.e., preterm birth), were included as the predictors for MICE to minimize the bias caused by the missingness of covariates. For imputed analysis for each indicator of corticosteroids, the number of imputed datasets (m) was determined by the formula: $Relative efficiency \left( RE \right)=\left( 1+\frac{\lambda}{m} \right)^{-1}$, where lambda is the proportion of incomplete cases, and RE was set to be 95%. The m was rounded up to the nearest integer. For each imputed dataset, the iteration number was 20. The results of the imputation show satisfied convergence. In each imputed dataset, we reran the primary analysis before pooling the estimates using the *miceadd* package in R.


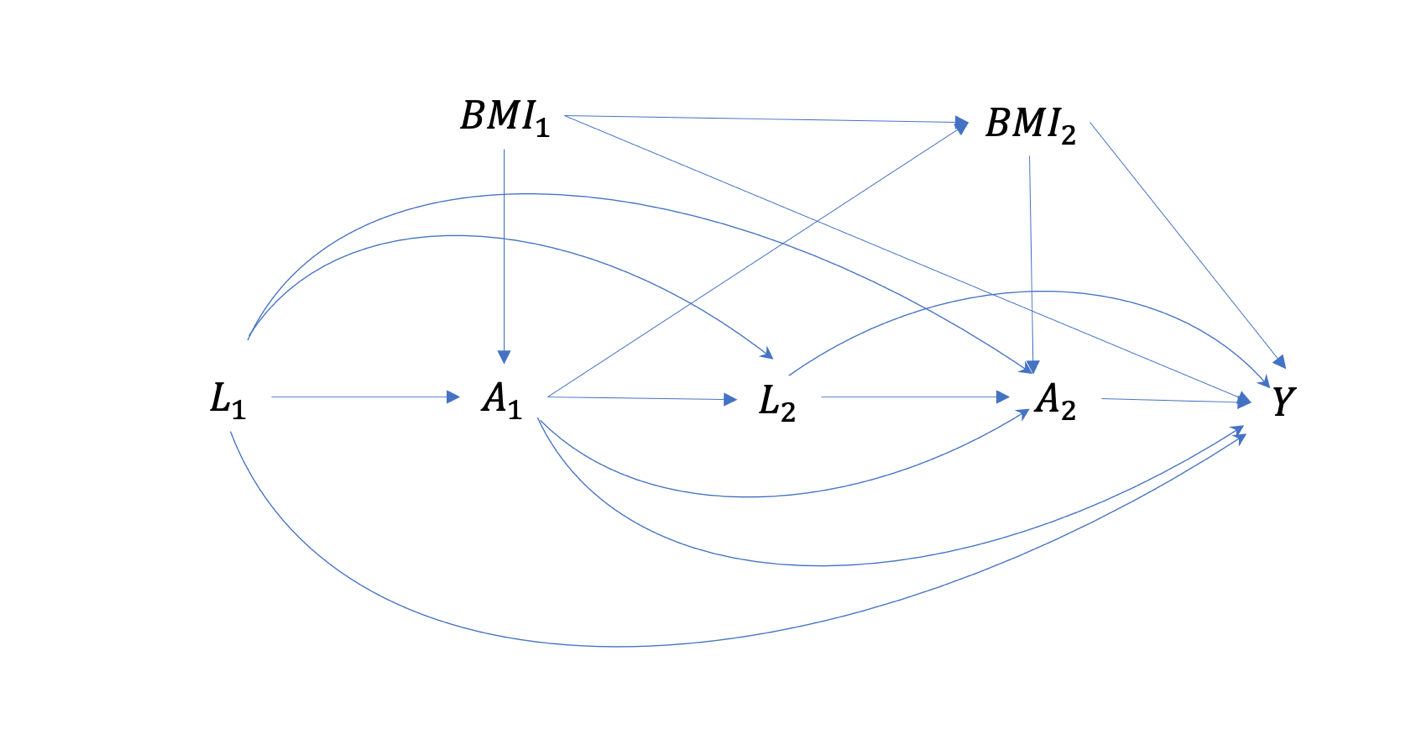


**Fig. S1**. The directed acyclic graph (DAG) describing the association among hair corticosteroids concentration, preterm birth, and selected covariates

A_1_: Hair corticosteroids concentration 0–3 months before pregnancy

A_2_: Hair corticosteroids concentration in the first trimester of pregnancy

L_1_: Maternal age, difficulty accessing basic foods, race, marital status, parity, gestational age at hair collection, hair treatment (hair tint, dye, and washing frequency)

L_2_: Employment during pregnancy, planned pregnancy, infant sex

BMI_1_: Body mass index (BMI) before pregnancy

BMI_2_: Body mass index (BMI) in the first trimester of pregnancy

Y: Preterm birth

**Table S2.** Variables used to construct the propensity score model for each occasion of hair corticosteroid concentration and formula of SIPW

| Exposure ($A$) | Potential confounders ($C$) | Exposure in the last occasion ($A$) | SIPW |
| --- | --- | --- | --- |
| Pre-pregnancy ($A_{1}$) | Maternal age, difficulty accessing basic foods, race, marital status, parity, gestational age at hair collection, hair treatment (hair tint, dye, and washing frequency), and body mass index (BMI) before pregnancy | - | $\frac{f_{A_{1}}\left( A_{1};\mu_{x},\sigma_{x}^{2} \right)}{f_{A_{1}}\left( A_{1}\vert C=c;\mu_{y},\sigma_{y}^{2} \right)}$ |
| First trimester ($A_{2}$) | Maternal age, difficulty accessing basic foods, race, marital status, parity, gestational age at hair collection, hair treatment (hair tint, dye, and washing frequency), employment during pregnancy, planned pregnancy, infant sex, and body mass index (BMI) in the first trimester of pregnancy | Pre-pregnancy ($A_{1}$) | $\frac{f_{A_{2}}\left( A_{2}\vert A_{1}=a_{1};\mu_{x},\sigma_{x}^{2} \right)}{f_{A_{2}}\left( A_{2}\vert C=c, A_{1}=a_{1};\mu_{y},\sigma_{y}^{2} \right)}$ |

**Table S3.** Characteristics of pregnant women by study and non-study population

| **Characteristics** | **Study population (*N* = 1,807)**  n (%)^a^ | **Non-study population (*N* = 3633)**  n (%) |
| --- | --- | --- |
| Maternal age (year), mean (SD) | 28.0 (6.2) | 28.1 (6.3) |
| Maternal age (year) category |  |  |
| 18–19 | 101 (5.6) | 203 (5.6) |
| 20–29 | 1,008 (55.8) | 2,012 (55.6) |
| 30–34 | 393 (21.8) | 745 (20.6) |
| ≥35 | 304 (16.8) | 659 (18.2) |
| Difficulty in paying for basics |  |  |
| No | 987 (54.9) | 1,874 (51.8) |
| Yes | 811 (45.1) | 1,742 (48.2) |
| Employment during pregnancy |  |  |
| Unemployed | 913 (50.6) | 1,946 (53.8) |
| Employed | 892 (49.4) | 1,672 (46.2) |
| Marital status |  |  |
| Others | 311 (17.3) | 678 (18.8) |
| Married or live with a partner | 1,491 (82.7) | 2,926 (81.2) |
| Nulliparity |  |  |
| No | 959 (53.2) | 1,872 (51.8) |
| Yes | 842 (46.8) | 1,743 (48.2) |
| Ethnicity |  |  |
| Not Mestizo | 354 (19.6) | 876 (24.2) |
| Mestizo | 1,450 (80.4) | 2,737 (75.8) |
| Planned pregnancy |  |  |
| Unplanned | 1,065 (59.3) | 2,140 (59.5) |
| Planned | 731 (40.7) | 1,458 (40.5) |
| Infant sex |  |  |
| Male | 908 (51.2) | 1,341 (50.0) |
| Female | 867 (48.8) | 1,342 (50.0) |
| Pre-pregnant BMI (kg/m^2^), mean (SD) | 25.5 (4.0) | 25.3 (4.1) |
| Pre-pregnant BMI category |  |  |
| <18.5 | 26 (1.4) | 36 (1.2) |
| 18.5–24.9 | 885 (49.2) | 1,598 (52.7) |
| 25.0-29.9 | 657 (36.6) | 1,027 (33.9) |
| ≥30 | 229 (12.7) | 370 (12.2) |
| BMI at pregnancy (kg/m^2^), mean (SD) | 25.7 (4.1) | 25.6 (4.2) |
| BMI category at pregnancy |  |  |
| <18.5 | 32 (1.8) | 69 (1.9) |
| 18.5–24.9 | 820 (45.7) | 1,712 (48.1) |
| 25.0-29.9 | 695 (38.8) | 1,298 (36.4) |
| ≥30 | 246 (13.7) | 483 (13.6) |
| Preterm birth (gestational age < 37 weeks) |  |  |
| No | 1,676 (92.8) | 2,521 (94.1) |
| Yes | 131 (7.2) | 159 (5.93) |

Abbreviations: BMI, body mass index; SD, standard deviation

^a^ The number of participants in each variable may not be identical to the total sample sizes due to missingness.


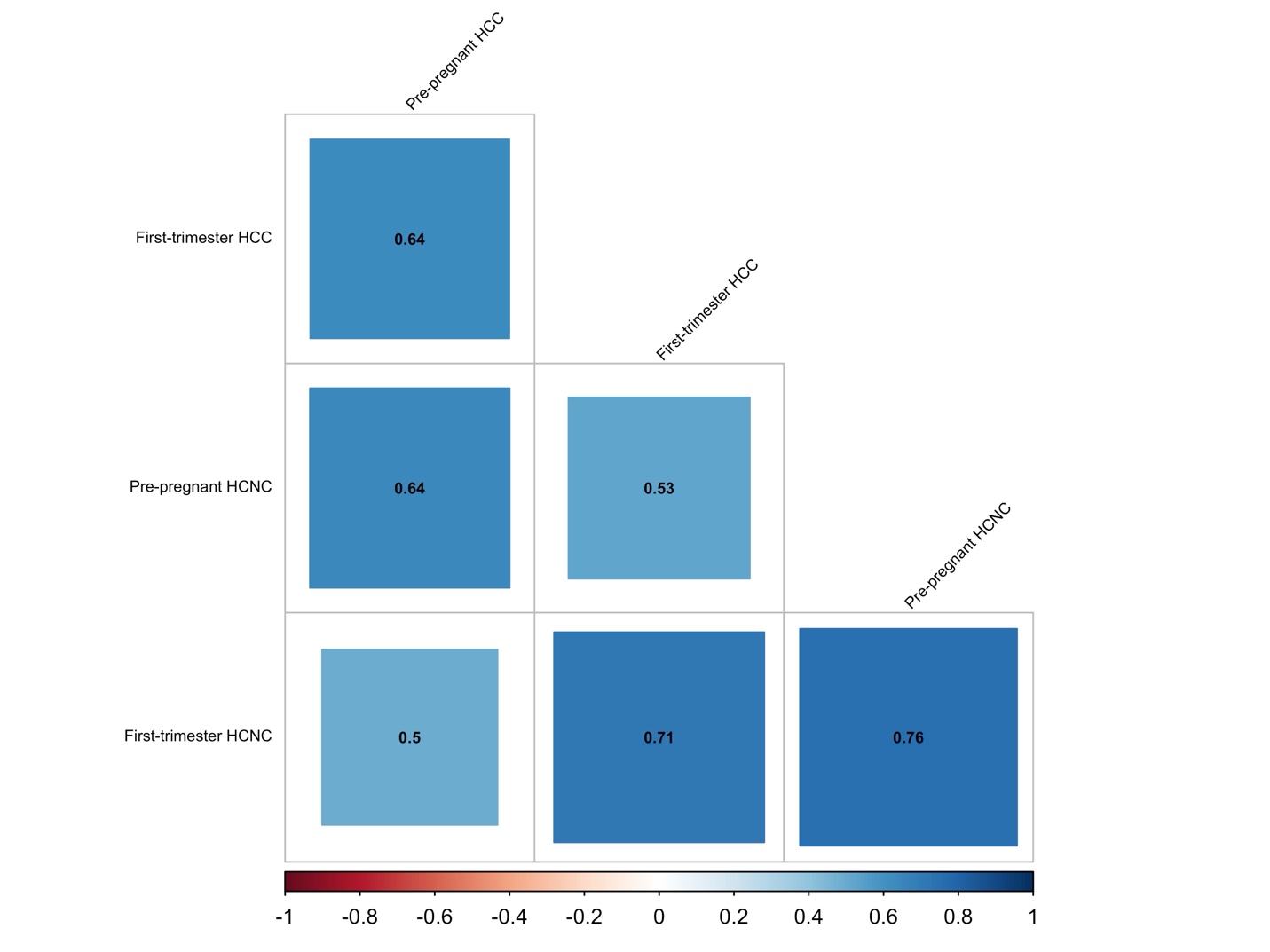
**Fig. S2.** The correlation between hair corticosteroid levels of pregnant women during pre-pregnancy and the first trimester of pregnancy

**Table S4.** Marginal association between hair corticosteroid levels and risk of preterm birth after multiple imputation

|  | Pre-pregnancy | First trimester |
| --- | --- | --- |
|  | RR (95%CI) | RR (95%CI) |
| Log HCC^a^  (*N* = 1739) | 0.91 (0.76,1.11) | 1.41 (1.16,1.71) |
| Log HCNC  (*N* = 1609) | 0.84 (0.61,1.16) | 1.23 (0.93,1.65) |

Abbreviation: HCC, hair cortisol concentration; HCNC, hair cortisone concentration.

^a^ Log HCC and Log HCNC have been standardized (mean = 0 and SD = 1).


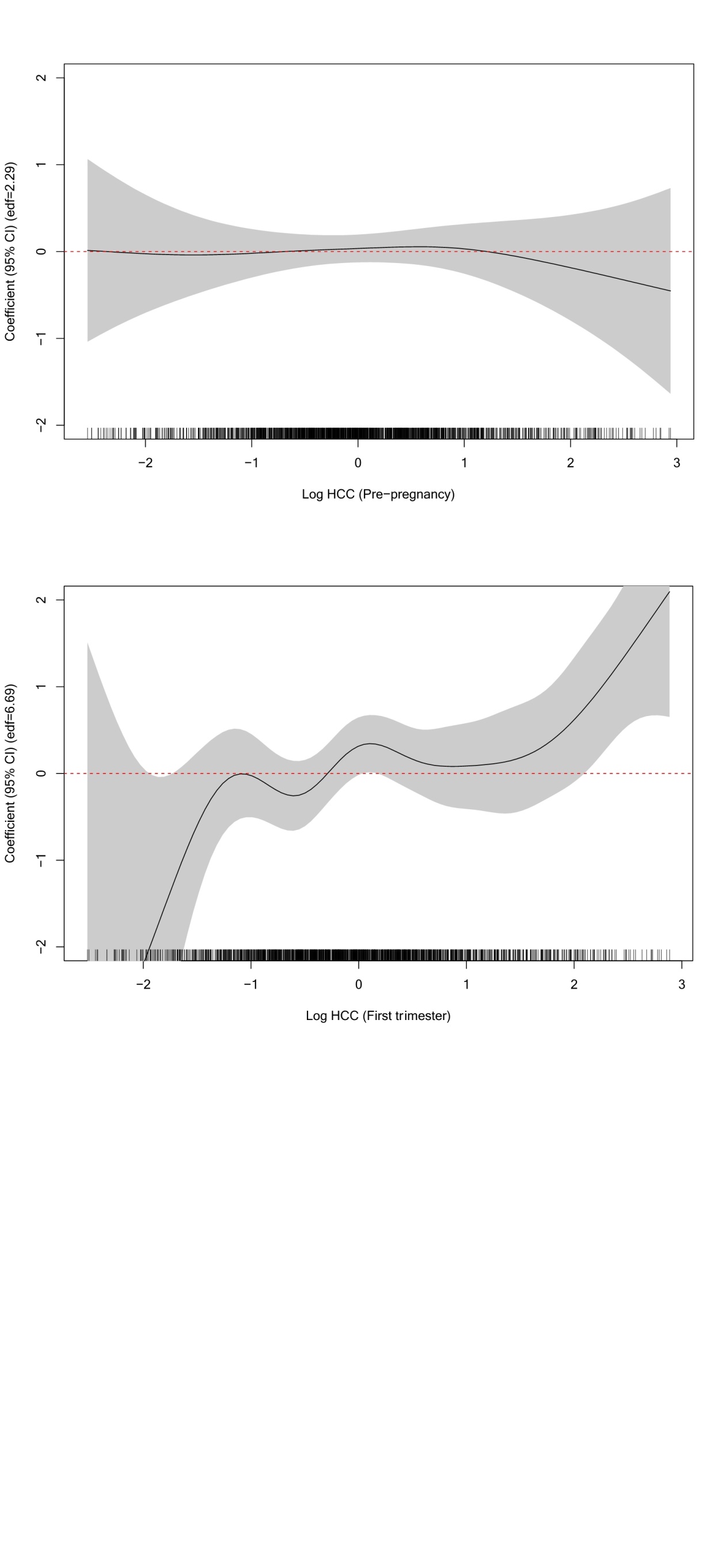


**Fig. S3.** Curvilinear pattern of the association between HCC and preterm birth risk (in Log scale)

**Abbreviation:** edf = effective degrees of freedom

**Note:** P for non-linearity = 0.13


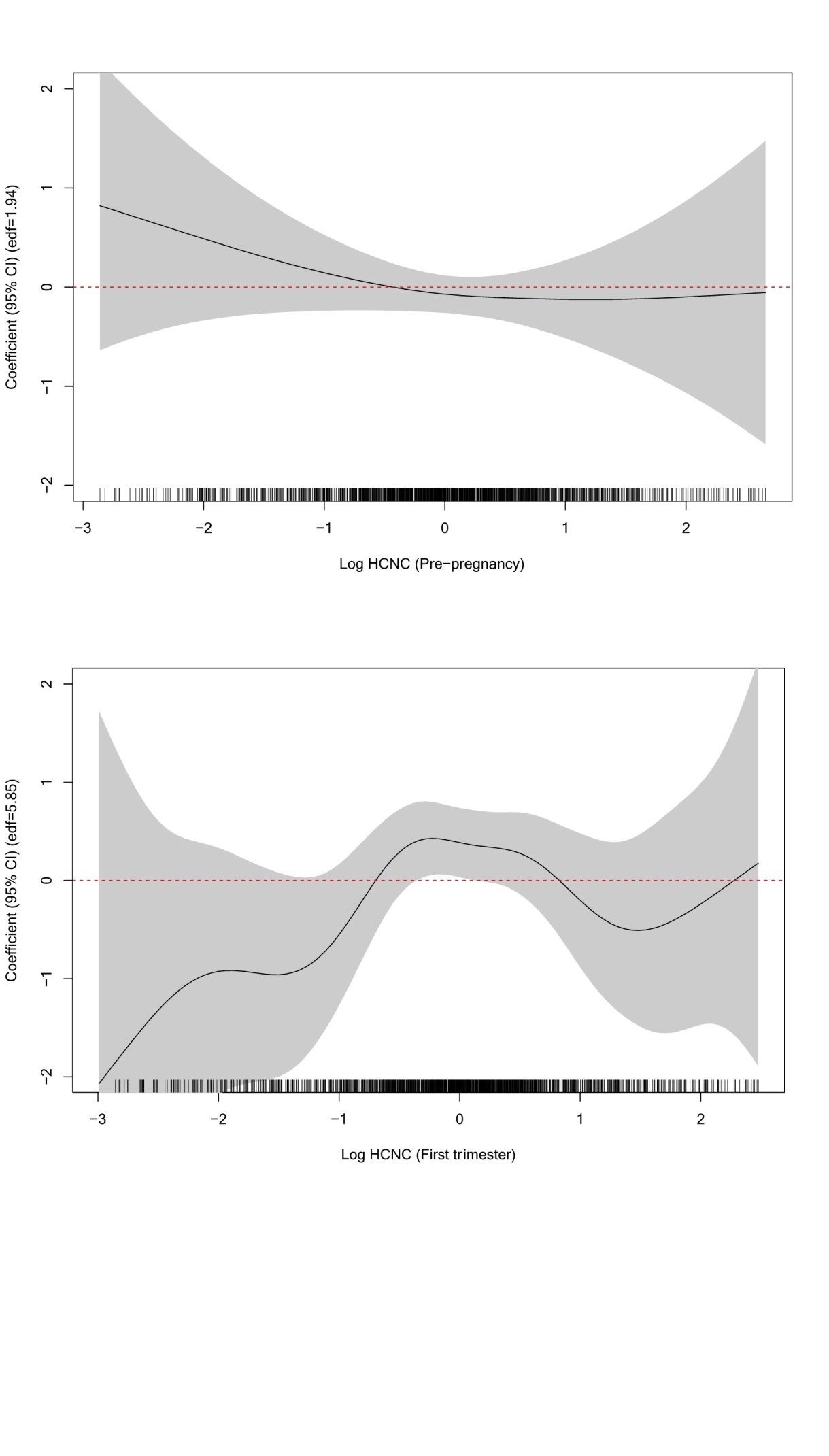


**Fig. S4.** Curvilinear pattern of the association between HCNC and preterm birth risk (in Log scale)

**Abbreviation:** edf = effective degrees of freedom

**Note:** P for non-linearity = 0.07

**Fig. S5.** Absolute correlation between Log HCC on different occasions and each potential confounder with and without SIPW.

**Fig. S6.** Absolute correlation between Log HCNC on different occasions and each potential confounder with and without SIPW.

**Table S5.** Marginal association between hair corticosteroid levels and risk of preterm birth (using multivariate linear regression to estimate propensity score)

|  | Pre-pregnancy | First trimester |
| --- | --- | --- |
|  | RR (95%CI) | RR (95%CI) |
| Log HCC^a^  (*N* = 1647) | 0.95 (0.77,1.16) | 1.39 (1.13,1.71) |
| Log HCNC  (*N* = 1520) | 0.84 (0.58,1.20) | 1.20 (0.87,1.65) |

Abbreviation: HCC, hair cortisol concentration; HCNC, hair cortisone concentration

^a^ Log HCC and Log HCNC have been standardized (mean = 0 and SD = 1).

**Table S6.** Weighted average absolute correlation between hair corticosteroid levels and each covariate using different approaches

|  | Pre-pregnancy | | First trimester | |
| --- | --- | --- | --- | --- |
|  | Weighted AAC (GAM) | Weighted AAC (LM) | Unweighted AAC (GAM) | Weighted AAC (LM) |
| Log HCC  (*N* = 1647) | 0.008 | 0.008 | 0.026 | 0.025 |
| Log HCNC  (*N* = 1520) | 0.013 | 0.013 | 0.048 | 0.046 |

Abbreviation: AAC, average absolute correlation; GAM, generalized additive model; HCC, hair cortisol concentration; HCNC, hair cortisone concentration; LM, linear regression

**Fig. S7.** Pairwise weighted absolute correlation between Log HCC on different occasions and each potential confounder by different approaches

**Fig. S8.** Pairwise weighted absolute correlation between Log HCNC on different occasions and each potential confounder by different approaches

**Table S7.** Marginal association between hair corticosteroid levels and risk of preterm birth (include serum C-reactive protein in the GPS model for the first trimester HCC and HCNC)

|  | Pre-pregnancy | First trimester |
| --- | --- | --- |
|  | RR (95%CI) | RR (95%CI) |
| Log HCC^a^  (*N* = 1624) | 0.97 (0.79,1.20) | 1.34 (1.08,1.67) |
| Log HCNC  (*N* = 1497) | 0.85 (0.59,1.23) | 1.17 (0.85,1.61) |

Abbreviation: HCC, hair cortisol concentration; HCNC, hair cortisone concentration

^a^ Log HCC and Log HCNC have been standardized (mean = 0 and SD = 1).
